# Advance Care Planning: Contemporary Issues and Future Directions

**DOI:** 10.1093/geroni/igx012

**Published:** 2017-08-28

**Authors:** Deborah Carr, Elizabeth A Luth

**Affiliations:** 1 Department of Sociology, Boston University, Massachusetts; 2 Department of Sociology, Rutgers University, New Jersey

**Keywords:** Advance care planning, Death and dying, Disparities, End of life, Epidemiology, Policy

## Abstract

Advance care planning (ACP) is widely considered an essential step toward achieving end-of-life care that is consistent with the preferences of dying patients and their families. ACP comprises a living will and a durable power of attorney for health care (DPAHC); these tools enable patients to articulate and convey their treatment preferences when they are still cognitively intact. In this article, we describe the strengths, weaknesses, and correlates of ACP in the United States, with attention to race and socioeconomic disparities therein. We then discuss other public policies and community programs designed to increase both the number of older adults who articulate their preferences for end-of-life care, and efficacy of ACP for ensuring that patients’ end-of-life treatment preferences are met. We describe the characteristics, strengths, and limitations of Physician Orders for Life Sustaining Treatment (POLSTs), and describe community programs, educational interventions, and public policies aimed toward increasing the prevalence and efficacy of end-of-life preparations. A key policy advance in the early 2010s has been Medicare coverage of one doctor–patient consultation session regarding end-of-life issues; we highlight the potentials and possible pitfalls of this policy. We conclude by identifying areas for future research, and highlighting practices from other nations that may help improve quality of end-of-life care in the United States.

**Translational Significance**

Advance care planning (ACP) is linked with greater use of palliative care among dying patients, lower medical expenditures at the end of life, and less distress among patients and their family members. Stark race and socioeconomic disparities in ACP rates have been documented, although recent policy advances such as Medicare reimbursement for doctor–patient consultation sessions and carefully targeted educational interventions and community programs may increase rates of planning among Blacks, Latinos, low-income adults, and others who may not have access to the professionals who encourage such preparations. The use of Physician Orders for Life Sustaining Treatment (POLSTs) may help to ensure greater clarity than living wills when conveying a dying patient’s treatment preferences to health care providers.

Dying is a late-life phenomenon. Of the 2.6 million deaths in the United States in 2016, roughly three-quarters were to older adults ages 65+ (Federal Interagency Forum on Aging Related Statistics, 2016). Late-life death typically follows a long-term chronic illness like cancer or heart disease, with most dying adults experiencing physical discomfort, functional decline, and cognitive impairment in the final weeks or months of life. Most older adults die in hospitals or nursing homes rather than at home, and many rely on medical technologies and aggressive treatments that increase the length—although not necessarily the quality—of their lives (Teno et al., 2013). Against this medical and technological backdrop, older adults often must make difficult decisions about the kinds of treatments they receive, so health care providers encourage them to convey their preferences for end-of-life care when they are still relatively healthy and cognitively intact (IOM, 2014). These preparations, widely referred to as advance care planning (ACP), are considered an essential step for achieving a “good death” in which physical pain and emotional distress are minimized, and the patient’s and family members’ treatment preferences are respected (Carr & Luth, 2016). ACP also is linked with reduced use of aggressive, costly and futile treatments at the end of life and greater use of palliative care, practices linked with both superior patient and survivor well-being (IOM, 2014). However, ACP has well-known practical limitations, leading policy makers to develop new practices such as Physician Orders for Life-Sustaining Treatment (POLSTs) (National POLST Paradigm, 2017a) and Medicare-reimbursed doctor-patient consultation sessions regarding end-of-life choices (Belluck, 2014). We describe the current state of knowledge on ACP, highlighting its strengths, limitations, correlates, and policies and programs designed to increase its effective use. We conclude by identifying avenues for future research, with an eye toward enhancing older Americans’ quality of death.

## Advance Care Planning: Background and Components

Dying patients and their caregivers frequently name patient involvement in end-of-life decision making as a core component of a “good death” or “dying well” (Byock, 1996; Steinhauser et al., 2000). Medical ethicists also emphasize the importance of patient autonomy, and propose that health care providers should share, and in some cases delegate, medical decision making to dying patients and their families (President’s Council on Bioethics, 2005). In practice, however, many dying older adults are unable to communicate their treatment preferences because they are incapacitated when the decision is required. An estimated 45%–70% of older adults facing end-of-life treatment decisions are incapable of making those decisions themselves (IOM, 2014). As such, difficult decisions about stopping or continuing treatment often fall upon family members who may be distressed or may disagree among themselves about an appropriate course of care (Kramer, Boelk, & Auer, 2006). When family members and health care providers cannot agree on a plan of action, the default decision is to continue treatments which may be financially and emotionally depleting for family members, and may compromise the patient’s quality of life.

In an effort to prevent problematic, futile, or contested end-of-life care, practitioners encourage adults to informally express and formally document their treatment preferences when they are still of sound mind and body (IOM, 2014). These efforts have been facilitated by the passage of the Patient Self-Determination Act (PSDA) in 1990; the Act requires all health care facilities receiving reimbursement from Medicare or Medicaid “to ask patients whether they have advance directives, to provide information about advance directives, and to incorporate advance directives into the medical record.” An advance directive typically comprises a living will and durable power of attorney for health care (DPAHC) designation. A living will articulates the treatments that an individual would want (or not want) at the end of life, such as ventilators, feeding tubes, or cardiopulmonary resuscitation (CPR). A DPAHC legally designates a specific individual (also referred to as a “surrogate” or “proxy”) to make decisions on behalf of the patient in the event that he or she is incapacitated (Carr & Luth, 2016). Most older adults select a spouse or long-term partner, followed by a child, or other close relative as DPAHC (Carr & Khodyakov, 2007). Spouses tend to be more knowledgeable than adult children regarding a patient’s preferences (Parks et al., 2011), with wives more knowledgeable than husbands (Zettel-Watson, Ditto, Danks, & Smucker, 2008).

Despite widespread professional endorsements, public awareness and education campaigns (Hammes, 2003), popular books (Gawande, 2014), and public policies (Patient Self-Determination Act of 1990, 1990) encouraging ACP, formal ACP has well-documented limitations. Criticisms of the living will include: the content may be unclear; one’s stated preferences may not be relevant to the patient’s condition, especially for dying older adults who drafted their living wills years earlier; and physicians may not have access to the document at the critical decision-making moment. Physicians also may be reluctant to follow the orders stated in the living will for fear of legal liability; in general, physicians believe their liability risk is greater if they do not attempt resuscitation than if they provide it against patient wishes (Burkle, Mueller, Swetz, Hook, & Keegan, 2012). Family members may not know or agree with the document’s content, or may not know how to translate vague preferences into specific clinical practices (Ditto et al., 2001).

DPAHC appointments also have practical limitations. Legally-appointed proxies have decision-making authority, yet may make decisions that create distress or disagreement among family members (Khodyakov & Carr, 2009). Surrogate decision makers’ knowledge of patient preferences is usually no better than chance (Shalowitz, Garrett-Mayer, & Wendler, 2006), with surrogates typically perceiving that the patient’s preferences mirror their own (Moorman & Inoue, 2013). Older adults might not inform the legal proxy of their preferences because they believe (erroneously) that their loved ones intuitively understand their preferences (Coppola, Ditto, Danks, & Smucker, 2001).

For some older patients, the proxy’s limited knowledge may be unproblematic; they may prefer that their family members do what they feel is best, rather than strictly follow the patient’s stated preferences (Moorman, 2011). Others may trust their physicians to make decisions for them (Su, 2008). Still, the patient’s deference to a specific decision maker’s wishes may create distress or conflict for concerned family members who do not hold decision-making power. Family members not designated as decision maker also may create difficulties; clinicians often encounter the “daughter in California” phenomenon, whereby a family member—especially one who resides far away from the dying patient and has had little engagement in a patient’s end-of-life care—enters the family conversation at the patient’s final stage of life. They try to undo, contest, undermine, or alter the decisions made by local family members who had been engaged in the patient’s care for a much longer duration. These family disagreements, in turn, may compromise health care providers’ ability to provide quality care (Kramer & Yonker, 2011). Given these well-documented limitations of formal ACP, some practitioners assert that informal conversations with significant others and care providers are the most critical component of end-of-life planning (Doukas & Hardwig, 2003). Discussions help to facilitate care consistent with the patient’s wishes; family members cannot adequately represent their dying relative’s preferences if they do not know what those preferences are.

Conversations about one’s general values are useful because few individuals know precisely how and of what cause they will die, making it difficult to specify particular interventions that they would want or not want at the end of life. A conversation about broad goals (e.g., “I do not want to be a vegetable”) and global preferences (e.g., “I do not want to be hooked up to machines”) may provide family members a general roadmap for representing their loved one’s wishes even in the absence of a formal living will articulating one’s specific plans for clinical care (Doukas & Hardwig, 2003). These conversations also may facilitate decision making in cases where the patient has not legally appointed a DPAHC. Most states have default systems for authorizing proxy decision makers. State laws vary, but these systems prioritize the immediate family—starting with spouse, followed by adult child, sibling, and other relatives (American Bar Association, 2009). Frank conversations about a patient’s values may empower and inform state-authorized proxies when making difficult decisions about their loved one’s care.

However, timing is critical, as some discussions may be “too little, too late.” Conversations regarding end-of life issues often are triggered by a patient’s health crisis such as a hospitalization or heart attack (Pfeifer, Mitchell, & Chamberlain, 2003). When discussions occur following triggering events, the patient (and family) may be too distressed to make an informed or appropriate decision about imminent care needs. Despite concerns regarding the efficacy and effectiveness of formal ACP tools, their use increases the chances of attaining some core components of a “good death,” including greater use of hospice or palliative care (Silveira, Kim, & Langa, 2010); reduced use of invasive or futile treatments such as feeding tubes or ventilators (Nicholas, Langa, Iwashyna, & Weir, 2011); a heightened sense of decision-making control (Edwards, Pang, Shiu, & Chan, 2010); a greater likelihood of dying at home rather than in an institution (Silveira et al., 2010); and fewer instances of receiving treatments discrepant with one’s wishes (Detering, Hancock, Reade, & Silvester, 2010).

Advance care planning also is associated with better outcomes for family members, including reduced decision-making burden, and fewer anxiety and depressive symptoms (Stein et al., 2013). Hospice use is an important pathway linking ACP with survivor well-being; bereaved family members whose loved one used hospice care have reduced risks of mortality, depression, and traumatic grief (Iwashyna & Christakis, 2003). However, even when the decedent had an advance directive in place, some family members may still report inadequate support during the dying process (Teno et al., 2007) or family conflict in cases where the living will was deemed unhelpful or problematic (Khodyakov & Carr, 2009).

Advance care planning is generally protective for both patients and family members, yet its impact on end-of-life medical expenditures is less clear. Studies based on large population-based samples generally show no significant effect (Kelley et al., 2011; Nicholas et al., 2011), whereas studies focused on specific disease groups such as advanced cancer patients suggest that ACP is linked with significantly reduced medical expenditures in the last six months of life (e.g., Zhang et al., 2009). Among older patients with advanced cancer, end-of-life medical costs are one-third less for persons who had an advance directive calling for limited care (Zhang et al., 2009). Intensive care unit (ICU) use is a key pathway linking ACP with reduced medical expenditures (Khandelwal et al., 2015). Researchers and policy makers agree that standard ACP tools cannot guarantee a good death, although their benefits outweigh their costs (IOM, 2014).

## ACP Trends and Differentials

ACP is intended to improve the quality of death for all Americans, yet in practice, it is largely undertaken by older adults of higher versus lower socioeconomic status (SES), Whites versus Blacks, and socially integrated versus isolated older adults. ACP rates also vary by age, marital and parental status, and other psychosocial factors. Only one-third to one-half of all adults in the United States have completed advance directives, although rates are as high as 70% among adults aged 65+ and persons with terminal illness (Carr & Moorman, 2009; Silveira et al., 2010). ACP rates have increased sharply since 1990; the proportion with a written advance directive more than doubled from 16% in 1990 to 35% in 2013 (Pew Research Center, 2013). Experts attribute this increase to the passage of the Patient Self-Determination Act (1990), prominent cases of contested end-of-life decisions such as that of Terri Schiavo, media programs like Bill Moyers’ PBS Series *On Our Own Terms,* best-selling books like Atul Gawande’s (2014)*Being Mortal,* and public awareness campaigns such as “Five Wishes” (Aging with Dignity, 2017). The Five Wishes, for example, is a user-friendly advance directive created by the nonprofit organization Aging with Dignity. The document can be obtained online, and is written in nontechnical language that includes identification of a proxy and preferences for medical and nonmedical treatment, comfort care, and one’s spiritual and interpersonal needs.

Rates of ACP are especially low among older Blacks and Latinos, relative to Whites. Estimates vary across samples, but most research finds that Whites are two to three times as likely as Blacks and Latinos to have an advance directive, with a much narrower gap for end-of-life discussions (Carr, 2011). However, these disparities have declined considerably over the past decade (Koss & Baker, 2017a). This narrowing may partly reflect cohort changes; one recent study of adults under age 64 found that race gaps in advance directive use were negligible (Carr, 2012a). Explanations for race disparities in ACP among current cohorts of older adults include: ethnic minorities’ limited access to medical and legal professionals who assist in preparing such documents; literacy or language barriers; beliefs that formal ACP is unnecessary because family members will make decisions collectively on behalf of the patient; historically rooted distrust of physicians and medical institutions; and adherence to religious beliefs that “God will decide” when it is time for a patient to die (Carr, 2011; 2012a; West & Hollis, 2012). Some Blacks and Latinos believe they do not need a living will because they desire all possible interventions at the end-of-life (Pew Research Center, 2013), and assume (incorrectly) that living wills limit rather than request treatment (Barnato, Anthony, Skinner, Gallagher, & Fisher, 2009; Mack et al., 2010).

This lack of ACP may prevent minority patients from receiving the treatments they desire. For example, among cancer patients who desire aggressive treatments, Blacks are one-third as likely as Whites to receive care that is consistent with their preferences (Loggers et al., 2009). Medical expenditures in the last six months of life are considerably higher for Blacks and Latinos, relative to Whites (Hanchate, Kronman, Young-Xu, Ash, & Emanuel, 2009). Fully 85 percent of these observed higher costs for Blacks and Hispanics are accounted for by their greater usage of intensive (and costly) invasive treatments. Thus, barriers to ACP among Blacks and Latinos are linked to costly intrusive treatments as well as the failure to receive desired treatments.

Very few studies examine SES differentials, yet emerging research shows that older adults with lower levels of education, income, assets, and home ownership rates are less likely than their more advantaged counterparts to do formal ACP, although no differences are found for discussions (Carr, 2012b). Older adults with assets to protect are more likely to do estate planning than their less wealthy counterparts. A visit to one’s lawyer to do complete one’s will often triggers the completion of related documents, including living wills and DPAHC appointments (Carr, 2012b). Low rates of estate planning also partly explain race disparities in ACP among older adults, and reflect racial disparities in wealth and home ownership (Koss & Baker, 2017b). Adults with lower levels of education and literacy also are less likely to do ACP, in part because they are reluctant to make decisions about treatments they don’t fully understand (Porensky & Carpenter, 2008; Waite et al., 2013).

Social relationships also shape ACP, such that people with supportive family relationships are more likely than those with troubled relationships to execute advance directives (Carr, Moorman, & Boerner, 2013). Psychological, religious, and attitudinal factors also may pose obstacles to ACP. Older adults with higher levels of death anxiety are less likely to do plan for end-of-life (Carr & Moorman, 2009). Those who have witnessed the painful or prolonged death of loved one are more likely to do ACP, perhaps in an effort to avoid a similar fate (Carr, 2012c). Religious beliefs also affect ACP; those who adhere to Fundamentalist beliefs (Sharp, Carr, & MacDonald, 2012), who believe that the length of their life is in God’s hands, who rate religion as “very important,” and whose religious beliefs guide their behavior are less likely to do formal ACP (Garrido, Idler, Leventhal, & Carr, 2013; Pew Research Center, 2013). Highly religious persons tend to desire all possible treatments at the end of life, because they believe that God will either sustain them or let them die “when the time is right” (Johnson, Kuchibhatla, & Tulsky, 2008).

Taken together, research shows stark race and socioeconomic disparities in formal ACP but not informal discussions – an activity that can be undertaken at no financial cost, and that does not require interactions with health care or legal professionals (Carr, 2012b). These patterns suggest that economic, informational, and structural barriers may be a more daunting obstacle to end-of-life planning among ethnic minorities and poorer adults than are cultural or attitudinal factors. These obstacles are potentially modifiable factors that may be addressed by innovative public policies, community initiatives and educational programs designed to place decision-making responsibility in the hands of older patients and their families, regardless of their personal resources. We next describe recent innovations to promote ACP, highlighting the extent to which these approaches may be effective in increasing ACP rates of all older adults, rather than just the most advantaged.

## Public Policy and Educational Interventions

### POLSTs (Physician Orders for Life Sustaining Treatment)

POLSTs (Physician Orders for Life Sustaining Treatment) offer a possible corrective to the limitations of living wills and DPAHC designations. They were introduced in Oregon in 1991 and are used in some format or are under development in 48 states as of 2017 (National POLST Paradigm, 2017a). POLSTs are completed by a patient in consultation with health care providers during the course of one’s clinical encounter. The document is signed by a physician; thus, health care providers are required to follow the content of the orders (National POLST Paradigm, 2017b). By contrast, a patient may complete a standard living will on their own or with their family or lawyer, without necessarily consulting their health care provider. Some patients fail to share their living will with their health care provider, instead leaving the document in a safe deposit box, the glovebox of their car, or even taped to their refrigerator. Because the POLST is completed during the clinical counter, it remains in the patient’s medical records (Yung, Walling, Min, Wenger, & Ganz, 2010).

POLSTs are appropriate for seriously ill individuals expected to die within 12 months (Hickman et al., 2011). POLSTs provide doctor’s orders regarding the specific treatments to be administered or withheld in medical scenarios that are common at the end of life, such as cardiopulmonary resuscitation (CPR) or feeding tubes (Schmidt, Zive, Fromme, Cook, & Tolle, 2014). The document also indicates whether a patient desires comfort measures only such as pain and breathing relief; limited interventions including antibiotics and intravenous (IV) fluids; or full treatment including intubation and mechanical ventilation (Schmidt et al., 2014).

POLSTs have several advantages over advance directives (for a full review, see Bomba, Kemp, & Black, 2012). Completion rates tend to be higher; studies of nursing home residents in four states show POLST completion rates are two to three times higher than advance directive completion rates, with no racial disparities evidenced (Hickman et al., 2011; Jennings et al., 2016). The widespread use of POLSTs may reflect the fact that they pose fewer obstacles to completion than living wills. Unlike living wills which must be executed by the patient, POLSTs can be completed by designated family members in consultation with the health care provider, if the patient is incapacitated (Hammes, Rooney, Gundrum, Hickman, & Hager, 2012). POLSTs are typically completed weeks before death, and reflect the current wishes of dying patients and their family, whereas advance directives often are completed months or even years before needed (Zive, Fromme, Schmidt, Cook, & Tolle, 2015). POLSTs also stipulate specific treatments that require little interpretation by practitioners (Hickman et al., 2011; Schmidt et al., 2014); empirical assessments show a 95 percent concordance rate between the care received at the end of life and the treatments articulated in the document (Hickman et al., 2011; Hammes et al., 2012). By contrast, studies have found weaker concordance for living wills, in part because doctors may be reluctant to withhold treatment; either because it is inconsistent with their training to treat and sustain life, or because they fear legal liability (Burkle et al., 2012). For example, one study of advanced cancer patients found that 13 percent received life-extending treatment in the last week of life despite having a stated preference for palliative or comfort care (Mack, Paulk, Viswanath, & Prigerson, 2010).

Despite these strengths, POLSTS have their limitations. Patients may not necessarily have high-quality conversations with their health care provider when completing the POLST together (Jennings et al., 2016). Although POLSTS were designed to eliminate some of the guess work of end-of-life decision making, the content may still require interpretation in the clinical setting. One analysis found that 10% of POLSTs have contradictory content, such as a patient completing a DNR yet also wanting full treatment (Schmidt et al., 2014). Given these potential contradictions, clinicians may misinterpret a POLST’s content and deny treatment that was desired (IOM, 2014). POLSTs also require a valid clinician’s signature; one recent study found that 1 in 10 POLSTs lacked this signature (Jennings et al., 2016).

### Medicare Coverage of ACP Consultation

In late 2015, the Centers for Medicare and Medicaid Services (CMS), the agency that runs Medicare, finalized regulations that allow Medicare to pay physicians and other qualified health care professionals for providing ACP consultation to beneficiaries. Prior to the implementation of this policy change on January 1, 2016, Medicare coverage rules allowed reimbursement for ACP consultation under very limited circumstances, focused largely on those with terminal illness. This partially explains why, prior to the policy’s implementation, only 27% of older adults had discussed their treatment preference with a physician, although 88% believe it is important to do so (Kaiser Family Foundation, 2015). This Medicare benefit may help to ensure that all older adults have the opportunity to discuss their treatment preferences with a health care provider, regardless of their economic or personal resources. These conversations also may help to minimize uncertainty and ambiguities in POLST completions. However, the Institute of Medicine (2014) urges careful monitoring of these consultation sessions to ensure that they are effective. Specifically, experts call for the development of quality of care metrics, and tying insurance reimbursement to these metrics. Ideally, these indicators would capture alignment among the patient’s goals, values, and preferences, the documented treatment plan, and the treatment ultimately delivered (IOM, 2014).

### Community-Based Programs

Community-based initiatives are a promising route for bringing end-of-life planning options to a broad base of older adults, often tailoring their programs to meet the distinctive social, cultural, religious, and language needs of the particular subpopulations they serve (see Figure 6-1, IOM, 2014 for detailed summary chart). One of the earliest community-based programs is Five Wishes, which was started by an attorney in the 1990s. For a small fee, users can obtain online a user-friendly, jargon-free template for completing an advance directive and naming a health care proxy (Aging with Dignity, 2017). A more recent development, The Conversation Project (2017), provides a free booklet in nine languages that offers tips for eliciting a patient’s values and structuring a meaningful discussion about end-of-life preferences. The Conversation Project, developed by the nonprofit Institute for Health Care Improvement, also provides resources on naming a DPAHC, discussing preferences with a physician, and facilitating decision making for individuals with dementia or Alzheimer ’s disease. Informational videos also are emerging as a widely used approach for raising awareness of and increasing rates of ACP. For example, ACP Decisions (2013) is a physician-led organization that provides educational videos on end-of-life care and medical decisions. The videos can be viewed for free on their website or licensed for use within health care organizations.

Promising new partnerships between health care systems and religious leaders are emerging with the explicit goal of increasing rates of end-of-life planning among older Blacks. For example, The Caring Touch Ministry, is a church-based hospice education and coordination program started by Cassandra Cotton, a church leader, certified nursing assistant (CNA), and community relations coordinator at a local hospice. The Ministry provides education on end-of-life issues to congregation members, including information on precisely what palliative care is, with careful attention to addressing myths regarding comfort care (NHPCO, 2015).

Community-based initiatives heighten awareness and trigger conversations about end-of-life care by providing accessible resources to the general population. However, such programs are rarely evaluated formally, making it difficult to determine their reach and impact. ACP Decisions has received funding from the National Institutes of Health to develop and evaluate their educational videos; these assessments show that hospitalized patients who watch the videos have more accurate knowledge about CPR and intubation and are more likely to complete POLSTs and to discuss ACP, relative to those who receive verbal instructions only about treatment options (El-Jawahri et al., 2015).

### Educational Interventions in Formal Care Settings

Many educational interventions have been developed and tested in institutional settings including long-term care facilities and hospitals. Intervention studies are developed with the explicit goal of identifying what type and form of information or incentive is most effective in encouraging ACP, and whether particular strategies are best suited for specific subpopulations including older adults with dementia, homeless adults, and those suffering from particular diseases such as cancer or end-stage renal disease (IOM, 2014). The most renowned and long-running program is Respecting Choices, which began in 1991 at Gundersen Medical Center in LaCrosse, Wisconsin. The program produces educational materials for patients; trains facilitators to discuss end-of-life issues with patients and prepare them for what lies ahead; and ensures that advance directives are available in patients’ electronic medical records. Nearly all (96%) persons who die in LaCrosse, WI have an advance directive, and the program has been replicated throughout the United States and abroad (Hammes et al., 2012).

Educational videos are emerging as a particularly popular and effective form of encouraging ACP. Professionally produced videos are an important advance because some clinicians, even those with the best intentions, may not feel adequately prepared to discuss end-of-life decision making with their patients. Video support tools are designed to encourage ACP by triggering informed conversations and helping patients to envision their future circumstances, think realistically about the choices they may face, and deliberate about those choices. Randomized control studies consistently show that educational videos increase rates of both formal ACP and patients’ knowledge about end-of-life issues. For instance, one study of older hospitalized end-stage renal disease (ESRD) patients in Hawaii found that ACP documentation rates increased from 3% to 40% pre- and postexposure to the video intervention (Volandes et al., 2016), while a study of older heart disease patients detected a significant increase in knowledge about end-of-life medical treatments following exposure to a video (El-Jawahri et al., 2016). (see Chan, Sim, Zimmermann, & Krzyzanowska, 2016 and Weathers et al., 2016 for review articles on end-of-life planning intervention studies).

## Directions for Future Research

Public conversations and empirical research on end-of-life care have flourished over the past two decades, yet important questions remain unanswered. We briefly suggest five main areas of research that we believe will be fruitful. First, future studies should delve more fully into the challenges faced by individuals in “nontraditional” families, including same-sex partnerships, cohabiting couples, living apart together (LAT) relationships, and reconfigured families in which at least one partner has children from a prior marriage. Given structural lags, public policies may not keep pace with social and cultural changes. State-level policies that bestow decision making in a particular order may mean that older adults who rely on someone other than a legal spouse or biological child may require more deliberate conversations about their end-of-life preferences.

Second, research has documented race and SES disparities in ACP, but has not delved into intersections therein. Emerging research suggests that for current cohorts of young and midlife adults, racial disparities in ACP may be disappearing (Carr 2012a; Koss & Baker, 2017a). This convergence may reflect secular increases in educational attainment, declining levels of religiosity, and other factors that are important sources of heterogeneity even within a single racial or ethnic group. We suspect that among future cohorts of older adults, socioeconomic disadvantage may be more powerful than race, religion, or cultural beliefs in impeding ACP.

Third, relatively little research evaluates the efficacy of end-of-life conversations, and whether particular types of information, interpersonal dynamics, institutional settings, or the timing of such conversations are linked with efficacy in conveying older adults’ treatment preferences. We encourage future studies to identify factors associated with meaningful and productive conversations among family members, clinicians, legal professionals, and others who may be instrumental in shaping older adults’ ACP. The recent CMS changes that reimburse health care providers for their end-of-life conversations with Medicare beneficiaries hold great promise in enhancing the quality of end-of-life care, yet this promise hinges on the quality of the conversations that ensue.

Fourth, we believe that POLSTs hold great potential for ensuring that dying adults’ treatment preferences are met, especially among populations that may lack the knowledge or connections that facilitate the use of more traditional ACP tools like living wills and DPAHC designations. However, further research is needed to demonstrate the efficacy of POLSTs, and to highlight those contexts in which they are most valuable. Assessments have focused largely on POLST utilization among nursing home residents (Hammes et al., 2012; Jennings, 2016), and less is known about usage rates and correlates thereof among community-dwelling older adults. We also believe that researchers should explore family members’ perceptions of the value, efficacy, and experience of using POLSTs. Although the primary goal of ACP is to enhance the quality of an older adult’s death, an equally important goal is to ease the decision-making process for family caregivers and to protect against bereaved family members’ symptoms of grief and distress, which are particularly prominent in the face of a “bad death” (Carr, 2003). The voices and perceptions of family caregivers who must live with a patient’s treatment decisions long after those decisions are carried out will be very valuable for scholars interested in enhancing the well-being of the millions of older adults who will inevitably outlive a family member.

Finally, international comparisons will help to identify relative strengths and weakness of ACP effectiveness and promotion efforts in the United States relative to other wealthy nations. ACP completion rates are considerably higher in the United States than other countries. An estimated 35% of the adult population ages 18+ in the United States have done ACP, compared to rates of less than 1% (Japan, Australia) to 7%–8% (Netherlands, UK) (Aw et al., 2011; Matsui, 2007; Pew Research Center, 2013; Stewart, 2005; Van Wijmen et al., 2010). Nonetheless, lessons for improving older adults’ quality of end-of-life care might be learned from considering alternative practices used in other nations. For example, despite relatively low rates of ACP completion, the United Kingdom was rated as providing the highest quality deaths out of 80 countries. By contrast, the United States ranked 9^th^ (The Economist Intelligence Unit, 2015). A key factor contributing to the United Kingdom’s superlative position is that its National Health Service (NHS) has developed and implemented a comprehensive framework for improving end of life care at the national and local levels. In addition to specific steps to facilitate more meaningful conversations between individuals and health care providers regarding end-of-life issues, additional facets include public support for a range of educational initiatives for chronically ill individuals and their caregivers, and providing palliative care training to all clinicians and staff involved in delivering end-of-life care (NHS Finance and Operations, 2016). A multipronged approach that targets not only ACP completion, but also patient and caregiver knowledge, and clinician training may be needed to enhance quality of end of life care for dying individuals.

## Conflict of Interest

None reported.

## References

[CIT0001] ACP Decisions (2013). Retrieved April 26, 2017 from https://www.acpdecisions.org/.

[CIT0002] Aging with Dignity (2017). Five Wishes resources. Retrieved May 3, 2107 from http://www.agingwithdignity.org/five-wishes.php.

[CIT0003] American Bar Association (2009). Default surrogate consent statutes. Retrieved May 2, 2017 from http://www.americanbar.org/content/dam/aba/migrated/aging/PublicDocuments/famcon_2009.authcheckdam.pdf.

[CIT0004] Aw D., Hayhoe B., Smajdor A., Bowker L. K., Conroy S. P., & Myint P. K (2011). Advance care planning and the older patient. QJM, 105, 225–230. doi:10.1093/qjmed/hcr209 22075012 10.1093/qjmed/hcr209

[CIT0005] Barnato A. E., Anthony D. L., Skinner J., Gallagher P. M., & Fisher E. S (2009). Racial and ethnic differences in preferences for end-of-life treatment. Journal of General Internal Medicine, 24, 695–701. doi:10.1007/s11606-009-0952-6 19387750 10.1007/s11606-009-0952-6 PMC2686762

[CIT0006] Belluck P (2014). Coverage for end-of-life talks gaining ground. New York Times (August 30, 2014). Retrieved November 21, 2014 from http://www.nytimes.com/2014/08/31/health/end-of-life-talks-may-finally-overcome-politics.html.

[CIT0007] Bomba P. A., Kemp M., & Black J. S (2012). POLST: An improvement over traditional advance directives. Cleveland Clinic Journal of Medicine, 79, 457–464. doi:doi:10.3949/ccjm.79a.11098 22751627 10.3949/ccjm.79a.11098

[CIT0008] Burkle C. M., Mueller P. S., Swetz K. M., Hook C. C., & Keegan M. T (2012). Physician perspectives and compliance with patient advance directives: The role external factors play on physician decision making. BMC Medical Ethics, 13, 31. doi:10.1186/1472-6939-13-31 23171364 10.1186/1472-6939-13-31 PMC3528447

[CIT0009] Byock I. R (1996). The nature of suffering and the nature of opportunity at the end of life. Clinics in Geriatric Medicine, 12, 237–252. 8799345

[CIT0010] Carr D (2003). A ‘good death’ for whom? Quality of spouse’s death and psychological distress among older widowed persons. Journal of Health and Social Behavior, 44, 215–232. 12866391

[CIT0011] Carr D (2011). Racial differences in end-of-life planning: Why don’t Blacks and Latinos prepare for the inevitable?Omega: Journal of Death & Dying, 63, 1–20. doi:10.2190/OM.63.1.a 21748919 10.2190/OM.63.1.a

[CIT0012] Carr D (2012a). Racial and ethnic differences in advance care planning: Identifying subgroup patterns and obstacles. Journal of Aging and Health, 24, 923–947. doi:10.1177/0898264312449185 22740168 10.1177/0898264312449185

[CIT0013] Carr D (2012b). The social stratification of older adults’ preparations for end-of-life health care. Journal of Health and Social Behavior, 53, 297–312. doi:10.1177/0022146512455427 22940813 10.1177/0022146512455427

[CIT0014] Carr D (2012c). “‘I don’t want to die like that...’: The impact of significant others’ death quality on advance care planning.”The Gerontologist, 52: 770–781. doi:10.1093/geront/gns051 22547085 10.1093/geront/gns051 PMC3495910

[CIT0015] Carr D., & Khodyakov D (2007). Health care proxies in later life: Whom do we choose and why?Journal of Health and Social Behavior, 48, 180–94. doi:10.1177/002214650704800206 17583273 10.1177/002214650704800206

[CIT0016] Carr D., & Luth E (2016). End of life planning and health care. Handbook of aging and the social sciences (pp. 375–394, 8^th^ ed).L. K. George & K. Ferraro (Eds.). New York: Academic Press.

[CIT0017] Carr D., & Moorman S (2009). End of life treatment preferences among the young-old: An assessment of psychosocial influences. Sociological Forum, 24, 754–778. doi:10.1111/j.1573-7861.2009.01135.x 21057589 10.1111/j.1573-7861.2009.01135.x PMC2971558

[CIT0018] Carr D., Moorman S., & Boerner K (2013). End-of-life planning in a family context: Does relationship quality affect whether (and with whom) older adults plan?Journal of Gerontology: Social Sciences, 68, 586–92. doi:10.1093/geronb/gbt034 10.1093/geronb/gbt034 PMC3674735 23689997

[CIT0019] Chan B., Sim H.-W., Zimmermann C., & Krzyzanowska M. K (2016). Systematic review of interventions to facilitate advance care planning (ACP) in cancer patients. Journal of Clinical Oncology, 34, 21–21. doi:10.1200/jco.2016.34.26_suppl.21

[CIT0020] The Conversation Project (2017). “Starter Kids.” Retrieved April 26, 2017 from http://www.theconversationproject.org/.

[CIT0021] Coppola K. P., Ditto P. H., Danks J. H., & Smucker W. D (2001). Accuracy of primary care and hospital-based physicians’ predictions of elderly outpatients’ treatment preferences with and without advanced directives. Archives of Internal Medicine, 161, 431–440. doi:10.1001/archinte.161.3.431 11176769 10.1001/archinte.161.3.431

[CIT0022] Detering K. M., Hancock A. D., Reade M. C., & Silvester W (2010). The impact of advance care planning on end of life care in elderly patients: Randomised controlled trial. British Medical Journal, 340, c1345. doi:10.1136/bmj.c1345 20332506 10.1136/bmj.c1345 PMC2844949

[CIT0023] Ditto P.H., Smucker W. D., Bookwala J., Coppola K.M., Dresser R., Fagerlin A.,…Zyzanski S (2001). Advance directives as acts of communication. Annals of Internal Medicine, 161, 421–30. doi:10.1001/archinte.161.3.421 10.1001/archinte.161.3.421 11176768

[CIT0024] Doukas D. J. & Hardwig J (2003). Using the family covenant in planning end-of-life care: Obligations and promises of patients, families, and physicians. Journal of American Geriatrics Society, 51, 1155–58. doi:10.1046/j.1532-5415.2003.51383.x 10.1046/j.1532-5415.2003.51383.x 12890082

[CIT0025] The Economist Intelligence Unit (2015). The 2015 quality of death index: Ranking palliative care across the world. Retrieved June 20, 2107 from http://www.eiuperspectives.economist.com/sites/default/files/2015%20EIU%20Quality%20of%20Death%20Index%20Oct%2029%20FINAL.pdf.

[CIT0026] Edwards A., Pang N., Shiu V., & Chan C (2010). Review: The understanding of spirituality and the potential role of spiritual care in end-of-life and palliative care: A meta-study of qualitative research. Palliative Medicine, 24, 753–770. doi:10.1177/0269216310375860 20659977 10.1177/0269216310375860

[CIT0027] El-Jawahri A., Mitchell S. L., Paasche-Orlow M. K., Temel J. S., Jackson V. A., Rutledge R. R.,…Lopez L (2015). A randomized controlled trial of a CPR and intubation video decision support tool for hospitalized patients. Journal of General Internal Medicine, 30, 1071–1080. doi:10.1007/s11606-015-3200-2 25691237 10.1007/s11606-015-3200-2 PMC4510229

[CIT0028] El-Jawahri A., Paasche-Orlow M. K., Matlock D., Stevenson L. W., Lewis E. F., Stewart G.,… & Temel J. S (2016). Randomized, controlled trial of an advance care planning video decision support tool for patients with advanced heart failure: clinical perspective. Circulation, 134, 52–60. doi:10.1161/CIRCULATIONAHA.116.021937 27358437 10.1161/CIRCULATIONAHA.116.021937 PMC4933326

[CIT0029] Federal Interagency Forum on Aging Related Statistics (2016). Older Americans 2016: Key Indicators of Well-Being. Washington, DC: Department of Health and Human Services.

[CIT0030] Garrido M. M., Idler E., Leventhal H. & Carr D (2013). Advance care planning: The role of end-of-life values and beliefs about control over the length of life. The Gerontologist, 53, 801–816. doi:10.1093/geront/gns128 23161430 10.1093/geront/gns128 PMC3771671

[CIT0031] Gawande A (2014). Being mortal: Medicine and what matters in the end. New York: Metropolitan Books.

[CIT0032] Hammes B. J (2003). Update on Respecting Choices ®: Four years on. Innovations in End-of-Life Care. Retrieved May 1, 2017 from http://www.rpctraining.com.au/module06/mod06_toolkit/Additional%20references/Reference-4-3.pdf.

[CIT0033] Hammes B. J., Rooney B. L., Gundrum J. D., Hickman S. E., & Hager N (2012). The POLST program: A retrospective review of the demographics of use and outcomes in one community where advance directives are prevalent. Journal of Palliative Medicine, 15, 77–85. doi:10.1089/jpm.2011.0178 22233467 10.1089/jpm.2011.0178

[CIT0034] Hanchate A., Kronman A. C., Young-Xu Y., Ash A. S., & Emanuel E (2009). Racial and ethnic differences in end of life costs: Why do minorities cost more than Whites?Archives of Internal Medicine, 169, 493–501. doi:10.1001/archinternmed.2008.616 19273780 10.1001/archinternmed.2008.616 PMC3621787

[CIT0035] Hickman S. E., Nelson C. A., Moss A. H., Tolle S. W., Perrin N. A., & Hammes B. J (2011). The consistency between treatments provided to nursing facility residents and orders on the physician orders for life-sustaining treatment form. Journal of the American Geriatrics Society, 59, 2091–2099. doi:10.1111/j.1532-5415.2011.03656.x 22092007 10.1111/j.1532-5415.2011.03656.x PMC3228414

[CIT0036] Institute of Medicine (IOM) (2014). Dying in America: Improving quality and honoring individual preferences near the end of life. Washington, DC: The National Academies. 25927121

[CIT0037] Iwashyna T. J. & Christakis N. A (2003). The health impact of health care on families: A matched cohort study of hospice use by decedents and mortality outcomes in surviving, widowed spouses. Social Science & Medicine, 57, 465–475. doi:10.1016/S0277-9536(02)00370-2 12791489 10.1016/s0277-9536(02)00370-2

[CIT0038] Jennings L. A., Zingmond D., Louie R., Tseng C. H., Thomas J., O’Malley K., & Wenger N. S (2016). Use of the physician orders for life-sustaining treatment among California nursing home residents. Journal of General Internal Medicine, 31, 1119–1126. doi:10.1007/s11606-016-3728-9 27188700 10.1007/s11606-016-3728-9 PMC5023600

[CIT0039] Johnson K. S., Kuchibhatla M., & Tulsky J. A (2008). What explains racial differences in the use of advance directives and attitudes toward hospice care?Journal of the American Geriatrics Society, 56, 1953–1958. doi:10.1111/j.1532-5415.2008.01919.x 18771455 10.1111/j.1532-5415.2008.01919.x PMC2631440

[CIT0040] Kaiser Family Foundation (2015). Public strongly favors end-of-life conversations between doctors and patients, with about eight in 10 saying Medicare and other insurers should cover these visits. Retrieved May 3, 2017 from http://www.kff.org/health-costs/press-release/public-strongly-favors-end-of-life-conversations-between-doctors-and-patients-with-about-eight-in-10-saying-medicare-and-other-insurers-should-cover-these-visits/.

[CIT0041] Kelley A. S., Ettner S. L., Morrison S., Du Q., Wenger N. S., & Sarkisian C. A (2011). Determinants of medical expenditures in the last 6 months of life. Annals of Internal Medicine, 154, 235–242. doi:10.7326/0003-4819-154-4-201102150-00004 21320939 10.7326/0003-4819-154-4-201102150-00004 PMC4126809

[CIT0042] Khandelwal N., Kross E. K., Engelberg R. A., Coe N. B., Long A. C., & Curtis J. R (2015). Estimating the effect of palliative care interventions and advance care planning on ICU utilization: A systematic review. Critical Care Medicine, 43, 1102–1111. doi:10.1097/CCM.0000000000000852 25574794 10.1097/CCM.0000000000000852 PMC4499326

[CIT0043] Khodyakov D., & Carr D (2009). The impact of late-life parental death on adult sibling relationships: Do parents’ advance directives help or hurt?Research on Aging, 31, 495–519. doi:10.1177/0164027509337193 20686628 10.1177/0164027509337193 PMC2914328

[CIT0044] Koss C. S., & Baker T. A (2017a). Race differences in advance directive completion: The narrowing gap between white and African American older adults. Journal of Aging and Health, 292, 324–342. doi:10.1177/0898264316635568 10.1177/0898264316635568 26944809

[CIT0045] Koss C. S., & Baker T. A (2017b). Where there’s a will: The link between estate planning and disparities in advance care planning by white and black older adults. Research on Aging. doi:10.1177/0164027517697116 10.1177/0164027517697116 29298597

[CIT0046] Kramer B. J. & Yonker J. A (2011). Perceived success in addressing end-of-life care needs of low-income elders and their families: What’s family conflict got to do with it?Journal of Pain and Symptom Management, 41, 35–48. doi:10.1016/j.jpainsymman.2010.04.017 20832980 10.1016/j.jpainsymman.2010.04.017

[CIT0046a] Kramer B.J., Boelk A.Z. and Auer C, (2006) Family conflict at the end of life: Lessons learned in a model program for vulnerable older adults. Journal of Palliative Medicine, 9, 791–801. doi:10.1089/jpm.2006.9.791 16752985 10.1089/jpm.2006.9.791

[CIT0047] Loggers E. T., Maciejewski P. K., Paulk E., DeSanto-Madeva S., Nilsson M., Viswanath K.,…Prigerson H (2009). Racial differences in predictors of intensive end of life care in advanced cancer patients. Journal of Clinical Oncology, 27, 5559–5564. doi:10.1200/JCO.2009.22.4733 19805675 10.1200/JCO.2009.22.4733 PMC2792953

[CIT0048] Mack J. W., Paulk E., Viswanath K., & Prigerson H. G (2010). Racial disparities in the outcomes of communication on medical care received near death. Archives of Internal Medicine, 170, 1533–1540. doi:10.1001/archinternmed.2010.322 20876403 10.1001/archinternmed.2010.322 PMC3739279

[CIT0049] Matsui M (2007), Perspectives of elderly people on advance directives in Japan. Journal of Nursing Scholarship, 39, 172–176. doi:10.1111/j.1547-5069.2007.00163.x 17535318 10.1111/j.1547-5069.2007.00163.x

[CIT0050a] Moorman S. M (2011). Older adults’ preferences for independent or delegated end-of-life medical decision making. Journal of Aging and Health, 23, 135–157. doi:10.1177/0898264310385114 20947875 10.1177/0898264310385114 PMC3060618

[CIT0050] Moorman S. M., & Inoue M (2013). Predicting a partner’s end of-life preferences, or substituting one’s own?Journal of Marriage and Family, 75, 734–745. doi:10.1111/jomf.12030

[CIT0051] National POLST Paradigm (2017a). Current state status. Retrieved July 28, 2017 from http://polst.org/programs-in-your-state/.

[CIT0052] National POLST Paradigm (2017b). POLST paradigm fundamentals. Retrieved April 25, 2017 from http://www.polst.org/wp-content/uploads/2016/09/POLST-Paradigm-Fundamentals.pdf.

[CIT0053a] NHPCO. (2015). NHPCO’s Facts and Figures (2015 Edition). Alexandria, VA: National Hospice and Palliative Care Organization Retrieved July 28, 2017 from https://www.nhpco.org/sites/default/files/public/Statistics_Research/2015_Facts_Figures.pdf.

[CIT0053] NHS Finance and Operations (2016). Our commitment to you for end of life care: The government response to the review of choice in end of life care. (July 2016). Retrieved June 20, 2017 from https://www.gov.uk/government/uploads/system/uploads/attachment_data/file/536326/choice-response.pdf.

[CIT0054] Nicholas L. H., Langa K. M., Iwashyna T. J., & Weir D. R (2011). Regional variation in the association between advance directives and end-of-life Medicare expenditures. Journal of the American Medical Association, 306, 1447–1453. doi:10.1001/jama.2011.1410 21972306 10.1001/jama.2011.1410 PMC3332047

[CIT0055] Parks S. M., Winter L., Santana A. J., Parker B., Diamond J. J., Rose M., & Myers R. E (2011). Family factors in end-of-life decision-making: Family conflict and proxy relationship. Journal of Palliative Medicine, 14, 179–184. doi:10.1089/jpm.2010.0353 21254816 10.1089/jpm.2010.0353 PMC3037808

[CIT0056] Patient Self-Determination Act of 1990 (1990). 554206, 4751 of the Omnibus Reconciliation Act of 1990. Pubs. No. 101–508.

[CIT0057] Pew Research Center (2013). Views on end-of-life medical treatments (November 2013). Retrieved May 2, 2017 from http://www.pewforum.org/files/2013/11/end-of-life-survey-report-full-pdf.pdf.

[CIT0058] Pfeifer M. P., Mitchell C.K., & Chamberlain L (2003). The value of disease severity in predicting patient readiness to address end-of-life issues. Archives of Internal Medicine, 163, 609–612. doi:10.1001/archinte.163.5.609 12622608 10.1001/archinte.163.5.609

[CIT0059] Porensky E. K., & Carpenter B. D (2008). Knowledge and perceptions in advance care planning. Journal of Aging and Health, 20, 89–106. doi:10.1177/0898264307309963 18252936 10.1177/0898264307309963

[CIT0060] President’s Council on Bioethics (2005). Taking care: Ethical caregiving in our aging society. Washington, DC: President’s Council on Bioethics.

[CIT0061] Schmidt T. A., Zive D., Fromme E. K., Cook J. N., & Tolle S. W (2014). Physician orders for life-sustaining treatment (POLST): Lessons learned from analysis of the Oregon POLST Registry. Resuscitation, 85, 480–485. doi:10.1016/j.resuscitation.2013.11.027 24407052 10.1016/j.resuscitation.2013.11.027

[CIT0062] Shalowitz D. I., Garrett-Mayer E., & Wendler D (2006). The accuracy of surrogate decision makers: A systematic review. Archives of Internal Medicine, 166, 493–497. doi:10.1001/archinte.166.5.493 16534034 10.1001/archinte.166.5.493

[CIT0063] Sharp S., Carr D., & MacDonald C (2012). Religion and end-of-life treatment preferences: Assessing the effects of religious denomination and beliefs. Social Forces, 91, 275–298. doi:10.1093/sf/sos061

[CIT0064] Silveira M. J., Kim S. Y. H., & Langa K. M (2010). Advance directives and outcomes of surrogate decision making before death. New England Journal of Medicine, 362, 1211–8. doi:10.1056/NEJMsa0907901 20357283 10.1056/NEJMsa0907901 PMC2880881

[CIT0065] Stein R. A., Sharpe L., Bell M. L., Boyle F. M., Dunn S. M., & Clarke S. J (2013). Randomized controlled trial of a structured intervention to facilitate end-of-life decision making in patients with advanced cancer. Journal of Clinical Oncology, 31, 3403–3410. doi:10.1200/JCO.2011.40.8872 23897967 10.1200/JCO.2011.40.8872

[CIT0066] Steinhauser K. E., Clipp E. C., McNeilly M., Christakis N. A., McIntyre L. M., & Tulsky J. A (2000). In search of a good death: Observations of patients, families, and providers. Annals of Internal Medicine, 132, 825–832. doi:10.7326/0003-4819-132-10-200005160-00011 10819707 10.7326/0003-4819-132-10-200005160-00011

[CIT0067] Stewart C (2005). The Australian experience of advance directives and possible future directions. Australasian Journal on Ageing, 24, S25–S29. doi:10.1111/j.1741-6612.2005.00099.x

[CIT0068] Su Y (2008). Looking beyond retirement: Patterns and predictors of formal end-of-life planning among retirement age individuals. Journal of Family and Economic Issues, 29, 654–73. doi:10.1007/s10834-008-9130-y

[CIT0069] Teno J. M., Gozalo P. L., Bynum J. P. W., Leland N. E., Miller S. C., Morden N. E.,…Mor V (2013). Change in end-of-life care for Medicare beneficiaries: Site of death, place of care, and health care transitions in 2000, 2005, and 2009. Journal of the American Medical Association, 309, 470–477. doi:10.1001/jama.2012.207624 23385273 10.1001/jama.2012.207624 PMC3674823

[CIT0070] Teno J. M., Gruneir A., Schwartz Z., Nanda A., & Wetle T (2007). Association between advance directives and quality of end-of-life care: A national study. Journal of the American Geriatrics Society, 55, 189–194. doi:10.1111/j.1532-5415.2007.01045.x 17302654 10.1111/j.1532-5415.2007.01045.x

[CIT0071] van Wijmen M. P., Rurup M. L., Pasman H. R., Kaspers P. J., & Onwuteaka-Philipsen B. D (2010). Advance directives in the Netherlands: An empirical contribution to the exploration of a cross-cultural perspective on advance directives. Bioethics, 24, 118–126. doi:10.1111/j.1467-8519.2009.01788.x 20136820 10.1111/j.1467-8519.2009.01788.x

[CIT0072] Volandes A. E., Paasche-Orlow M. K., Davis A. D., Eubanks R., El-Jawahri A., & Seitz R (2016). Use of video decision aids to promote advance care planning in Hilo, Hawaii. Journal of General Internal Medicine, 31, 1035–1040. doi:10.1007/s11606-016-3730-2 27194151 10.1007/s11606-016-3730-2 PMC4978682

[CIT0073] Waite K. R., Federman A.D., McCarthy M., Sudore R., Curtis L. M., Baker D. W.,…Paasche-Orlow M. K (2013). Literacy and race as risk factors for low rates of advance directives in older adults. Journal of the American Geriatrics Society, 61, 403–406. doi:10.1111/jgs.12134 23379361 10.1111/jgs.12134 PMC3891653

[CIT0074] Weathers E., O’Caoimh R., Cornally N., Fitzgerald C., Kearns T., Coffey A.,…Molloy D. W (2016) Advance care planning: A systematic review of randomised controlled trials conducted with older adults. Maturitas, 91, 101–109. doi:10.1016/j.maturitas.2016.06.016 27451328 10.1016/j.maturitas.2016.06.016

[CIT0075] West S. K., & Hollis M (2012). Barriers to completion of advance care directives among African Americans ages 25–84: A cross-generational study. Omega, 65, 125–137. doi:10.2190/OM.65.2.c 22953509 10.2190/OM.65.2.c

[CIT0076] Yung V. Y., Walling A. M., Min L., Wenger N. S., & Ganz D. A (2010). Documentation of advance care planning for community-dwelling elders. Journal of Palliative Medicine, 13, 861–867. doi:10.1089/jpm.2009.0341 20618087 10.1089/jpm.2009.0341 PMC2939845

[CIT0077] Zettel-Watson L., Ditto P. H., Danks J. H., & Smucker W. D (2008). Actual and perceived gender differences in the accuracy of surrogate decisions about life-sustaining medical treatment among older spouses. Death Studies, 32, 273–290. doi:10.1080/07481180701881230 18705171 10.1080/07481180701881230

[CIT0078] Zhang B., Wright A. A., Huskamp H. A., Nilsson M. E., Maciejewski M. L., Earle C. C.,…Prigerson H. G (2009). Health care costs in the last week of life: Associations with end-of-life conversations. Archives of Internal Medicine, 169, 480–488. doi:10.1001/archinternmed.2008.587 19273778 10.1001/archinternmed.2008.587 PMC2862687

[CIT0079] Zive D. M., Fromme E. K., Schmidt T. A., Cook J. N., & Tolle S. W (2015). Timing of POLST form completion by cause of death. Journal of Pain and Symptom Management, 50, 650–658. doi:10.1016/j.jpainsymman.2015.06.004 26162508 10.1016/j.jpainsymman.2015.06.004

